# Current clinical standards for renal transplantation: a survey among urological and surgical transplantation centers in Germany

**DOI:** 10.1007/s00345-025-06094-2

**Published:** 2025-11-20

**Authors:** Laura Müller, Hendrik Apel, Robert Peters, Frank Friedersdorff, Karoline Kernig, Philip Zeuschner, Michael Stöckle, Juliane Putz, Johannes Huber, Luka Flegar

**Affiliations:** 1 Department of Urology, Philipps University of Marburg, 35043 Marburg, Germany; 2 Department of Urology and Pediatric Urology, University Hospital Erlangen, Friedrich-Alexander-Universität Erlangen-Nürnberg, 91056 Erlangen, Germany; 3 Department of Urology, Charité-Universitätsmedizin Berlin, Corporate Member of Freie Universität Berlin, Humboldt-Universität zu Berlin and Berlin Institute of Health, 10117 Berlin, Germany; 4 Department of Urology, University Rostock, 18055 Rostock, Germany; 5 Department of Urology and Pediatric Urology, Saarland University, 66421 Homburg/Saar, Germany; 6 Department of Urology, University Hospital Carl Gustav Carus, Technische Universität Dresden, 01307 Dresden, Germany; 7 Department of Urology, University Hospital Heidelberg, 69117 Heidelberg, Germany

**Keywords:** Kidney transplantation, Standards, Survey, Dialysis, Perioperative treatment

## Abstract

**Purpose:**

Kidney transplants in Germany are either performed by surgical, urological or interdisciplinary teams. Hence, perioperative details might differ in relevant ways. The aim of our survey was to record and compare the respective perioperative standards.

**Methods:**

A structured 50-item questionnaire was sent via email to all certified German kidney transplant centers (*n* = 38). Anonymized data collection was performed with www.surveymonkey.com from November 2023 – May 2024.

**Results:**

Complete responses were obtained from 34/38 centers (response rate 89.5%); 13 were urological-led and 21 surgical-led programs. In most cases (76% urological vs. 90% surgical, *n* = 10 and 19), transplantation is performed extraperitoneally via hockey-stick incision. A peritoneal fenestration for lymphocele prophylaxis is not performed routinely (69% vs. 86%, *n* = 9 and 18). Vascular anastomoses are primarily frequently sutured with 5 − 0 or 6 − 0 prolene, and the ureteral anastomosis with 4 − 0, 5 − 0 or 6 − 0 PDS suture. The Lich-Gregoir technique is most commonly used for ureteral implantation (84% in urology vs. 76% in surgery, *p* = 0,55). There are only small differences in postoperative management between urological and surgical programs, e.g. regarding the duration of catheterization (7–10 days vs. < 7 days; *p* = 0.008) or the time of discharge (10–14 days vs. 6–8 days; *p* = 0,05).

**Conclusion:**

The majority of kidney transplants in Germany is performed according to similar standards. There are minor differences in the specialists approach, e.g. in the choice of suture material. Certain practices, such as peritoneal fenestration for lymphocele prophylaxis, warrant further scientific evaluation.

**Supplementary Information:**

The online version contains supplementary material available at 10.1007/s00345-025-06094-2.

## Introduction

Kidney transplantation has been an established surgical therapy for end-stage kidney disease since the mid-20th century [1]. Approximately 100,000 kidneys are transplanted worldwide annually [2]. In Germany, the responsibility for the surgical aspect of the treatment has historically fallen to both urologists and general surgeons. There are currently 38 kidney transplant centers in Germany.

The aim of this study was to investigate whether there is a difference in the surgical approach to kidney transplantation depending on whether the surgeon is trained as a urologist or a general surgeon. What is the standard for this highly specialized procedure in Germany?

## Materials and methods

### Survey

A structured 50-item questionnaire was emailed to all certified German kidney transplant centers (*n* = 38). The complete questionnaire is provided in the supplement.

Anonymized data collection took place with www.surveymonkey.com from November 2023 - May 2024. Response collection was closed on June 01 2024.

### Data analysis and assessment

A separate evaluation was carried out for urological vs. general surgical centers. Chi square tests were used for group comparison, with statistical significance set at α = 0.05. The questionnaire results are presented in a descriptive statistical manner using bar charts and pie charts. Statistical analyses were performed using R version 4.3.3 (R Foundation for Statistical Computing, Vienna, Austria).

When evaluating the questionnaires received, individual questionnaires were found to be incomplete. Missing data was counted as “not specified”. The questions that were answered in incomplete questionnaires were included in the evaluation. For presenting the results, we focus on the most relevant perioperative differences between urology-led and surgery-led kidney transplant centers. The complete summary of all responses can be found in the supplement.

## Results

### Participants characteristics

**Fig. 1:**
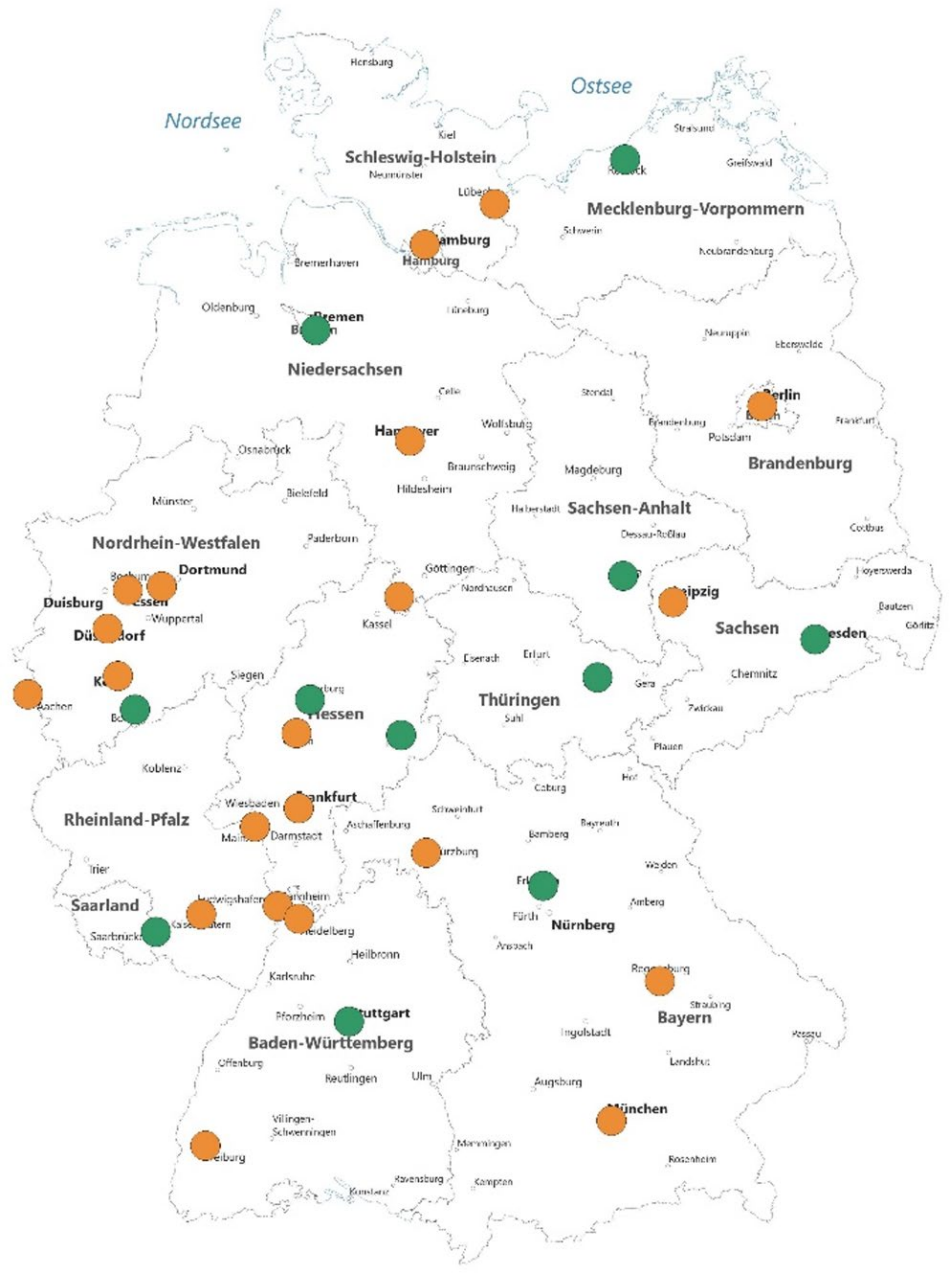
Participating kidney transplant centers; green marking for the urologically led programs and yellow marking for the surgically led programs

We received responses from 34/38 centers (response rate 89.5%); 13 were urological (38%) and 21 surgical programs (62%). Figure 1 displays the participating centers; with green indicated for the urology-led programs and yellow indicating surgery-led programs.

All respondents confirmed that they perform post-mortem kidney transplantation as well as living kidney donations. Pediatric kidney transplants are also performed in 45% and 39% of the urological and surgical centers, respectively. Details are shown in Table 1.

**Table 1:** Summary of differences between urologically and surgically led transplant centers

	Urological centers	Surgical centers	All	*P*-Value
Response rate	13/13 (100%)	21/25 (84%)	34/38 (89.5%)	
Post-mortem Tx	13 (100%)	21 (100%)	34 (100%)	
Living kidney donations	13 (100%)	21(100%)	34 (100%)	
Pediatric kidney transplantation	5 (45%)	7 (39%)	12 (35%)	0.70
Pre-operative:				
No Nicotine cessation	10 (91%)	11 (69%)	21 (78%)	0.17
No Weight reduction	7 (53%)	3 (14%)	10 (41%)	0.10
No Maximum BMI	7 (53%)	3 (14%)	10 (41%)	0.02 *
Perioperative:				
surgical approach via hockey stick incision	10 (76%)	19 (90%)	29 (85%)	0.28
surgical approach robotic-assisted laparoscopic	3 (23%)	1 (5%)	4 (12%)	0.10
surgical approach for donor organ retrieval minimally invasively	5 (38%)	15 (71%)	20 (59%)	0.02 *
lymphocele prophylaxis via peritoneal fenestration	3 (23%)	1 (5%)	4 (12%)	0.11
Suture material for vascular anastomoses: 5 − 0/6 − 0 prolene	9 (69%)	16 (76%)	25 (%)	0.69
Ureteral implantation according to Lich-Gregoir	11 (84%)	16 (76%)	27 (%)	0.55
Post-operative:				
intermediate care ward	6 (46%)	6 (29%)	12 (35%)	0.31
interdisciplinary post-operative treatment	1 (8%)	10 (48%)	11 (32%)	0.008 *
duration of catheterization < 7 days	2 (15%)	13 (62%)	15 (44%)	0.008 *
length of hospital stay 10–14 days	10 (77%)	9 (42%)	19 (56%)	0.04 *
early discharge (< 10 days)	0	5 (24%)	5 (15%)	0.05 *
Complications:				
Insertion or change of ureteral stent in case of urinary tract dilation	8 (61%)	12 (57%)	20 (59%)	0.89
Drainage in case of symptomatic lymphoceles	7 (53%)	9 (43%)	16 (47%)	0.58
ureteral reimplantation in case of symptomatic urinary reflux	4 (30%)	12 (57%)	16 (47%)	0.04 *
Individualized approach in case of symptomatic urinary reflux	4 (30%)	1 (5%)	5 (15%)	0.05 *

Arithmetical differences due to the exclusion of incomplete questionnaires

### Pre-operative standards

Most centers reported similar pre-operative patient preparation. Both nicotine cessation and weight normalization are not mandatory for the majority of urological surgeons surveyed (91% and 63%). In contrast, only 31% of general surgeons do not require weight reduction. However, this difference is not statistically significant (*p* = 0.10).

In addition, 53% of the urological centers (*n* = 7) do not have a maximum BMI limit for transplant candidates; the same applies to only 14% (*n* = 3) of the surgical centers (*p* = 0.02).

### Perioperative standards

The majority of kidney transplants are performed as open surgery, extraperitoneally via hockey stick incision. In the urologically managed centers, this is 76% (*n* = 10) and in the surgically managed centers even 90% (*n* = 19). However, a minority of 23% of urological transplant centers (*n* = 3) also perform robotic-assisted laparoscopic kidney transplants. Of the surgical-led transplant centers, only 5% do so (*n* = 1). This difference is not statistically significant (*p* = 0.10).

Donor organ retrieval for living kidney transplantation is predominantly performed via a minimally invasively approach (laparoscopically or retroperitoneoscopically). However, this is more common in surgical centers (*n* = 5, 38% vs. *n* = 15, 71%; *p* = 0.02).

Peritoneal fenestration for lymphocele prophylaxis is not performed in the majority of cases (69% and 86% of the urological and surgical centers, respectively).

**Fig. 2:**
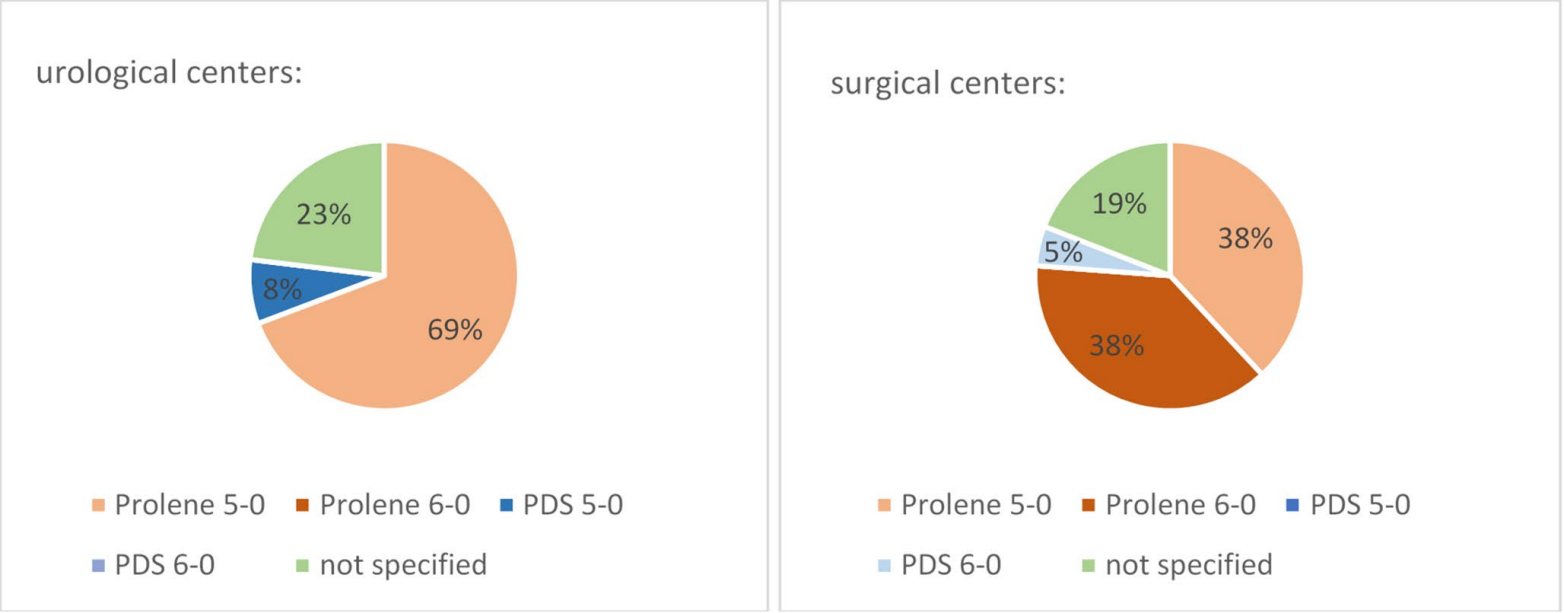
Sutures used for vascular anastomosis

As depicted in Fig. 2, vascular anastomoses are performed in 69% (*n* = 9) and 76% (*n* = 16) with 5 − 0 or 6 − 0 prolene in urological and surgical centers. Urologists tend to prefer continuous suturing to the front and back (61%, *n* = 8), and only rarely used the parachute technique (*n* = 1) whereas general surgeons use parachute technique more often at 43% (*n* = 9). However, the majority here also performs continuous anastomoses from front and back (57%, *n* = 12), which is a statistically significant increase (*p* = 0.04).

Ureteral anastomosis is performed in 46% of urologic centers with 5 − 0 PDS (*n* = 6) and in 23% with 5 − 0 Monocryl (*n* = 3). In contrast 62% of surgical centers use 4 − 0, 5 − 0 or 6 − 0 PDS (*n* = 13, *p* = 0.06). Ureteral implantation is performed in 84% of urological centers according to Lich-Gregoir technique (*n* = 11) vs. 76% in surgical centers (*n* = 16, *p* = 0.55).

**Fig. 3:**
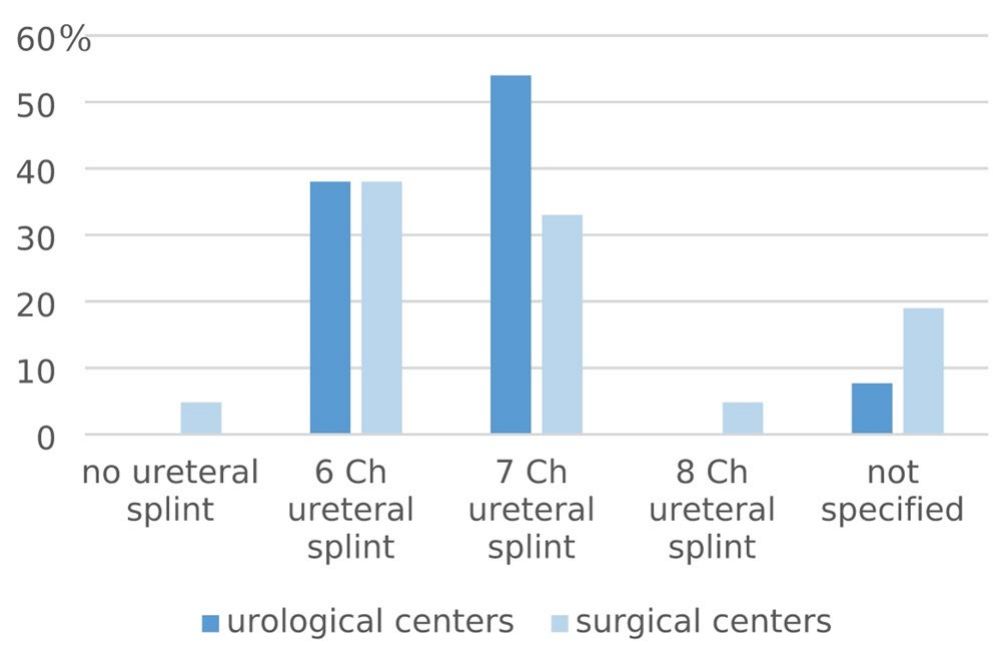
Intraoperative use of a ureteral stent

**Fig. 4:**
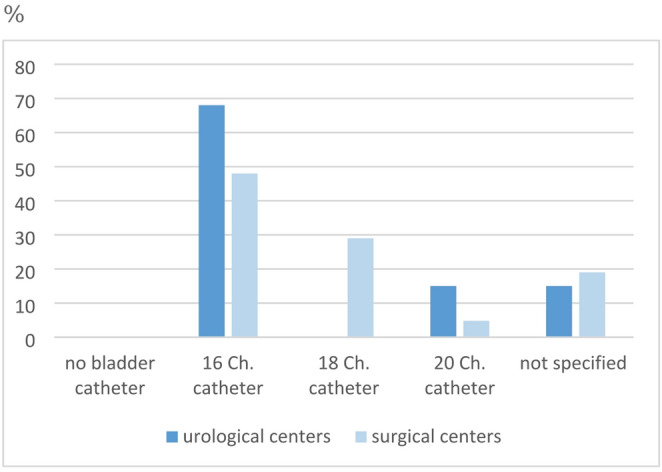
Intraoperative use of a bladder catheter

85% (*n* = 11) and 76% (*n* = 16) of urology and general surgery centers respectively insert a perioperative wound drainage. In almost all centers, a 6 or 7 Ch. ureteral stent as well as a transurethral bladder catheter are inserted. Figures 3 and 4 show further details regarding the use of ureteral stents and catheters.

### Post-operative standards

**Fig. 5:**
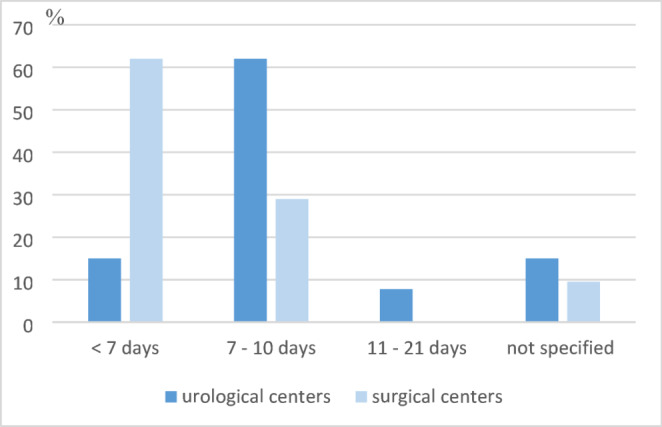
Duration of catheterization

In most transplant centers, postoperative care is provided on an intermediate care ward (46% and 29%, *n* = 6 each). In 46% of the urologically managed programs, the operating department itself is in charge of postoperative treatment (*n* = 6), while in 48% of the surgically managed programs there is an interdisciplinary concept (*n* = 10; *p* = 0.008). In the majority of urological centers, the bladder catheter is removed after 7–10 days (61%, *n* = 8). In most surgical centers, the removal takes place significantly earlier; after < 7 days (62%, *n* = 13; *p* = 0.008; Fig. 5).

Most patients are discharged after kidney transplantation on day 10–14 after surgery. This applies to 77% (*n* = 10) of urological and 42% (*n* = 9) of surgical patients. However, a significant proportion of the surgically managed clinics discharge their patients earlier (6–8 days, *p* = 0.05).

### Complications

A uniform picture emerges regarding the treatment of possible complications: in cases of urinary tract dilation in the transplanted kidney, 61% of urological clinics (*n* = 8) and 57% of surgical clinics (*n* = 12) insert or change the ureteral stent.

Symptomatic lymphoceles are managed with drainage in 53% (*n* = 7) and 43% (*n* = 9) of cases, respectively. Additionally, 23% (*n* = 3) and 33% (*n* = 7) of urological and surgical centers recommend minimally invasive lymphocele fenestration.

Surgical and urological centers differ in the treatment of symptomatic urinary reflux: while 57% of surgical centers perform ureteral reimplantation to treat reflux (*n* = 12), only 30% of urological centers do so (*n* = 4; *p* = 0.04). The latter recommend an individualized approach more frequently (30%, *n* = 4).

## Discussion

Despite the complexity of kidney transplantation and the involvement of different surgical specialties our study shows a fairly homogeneous picture of pre-, peri- and postoperative procedures across German transplantation centers. Perioperative standards are largely consistent across kidney transplant centers in Germany. This is particularly welcome against the background of quality improvement in surgical activities. The EAU Guideline on Renal Transplantation partially covers the demand for information on state-of-the-art treatments and standardization within this special group of patients [3].

Nonetheless, there are a few differences between the urologically-led and surgically-led programs: Regarding preoperative patient selection, there is a statistically non-significant but noticeable tendency with surgery-led programs being stricter regarding overweight patients undergoing surgery. Even though several studies in recent years have shown that kidney transplantations can also be performed safely in severely overweight patients, an increased perioperative risk is evident in this population [4, 5]. However, despite the risks, kidney transplantation in obese patients still provides a survival advantage over remaining on dialysis [6]. In the future, robotic-assisted kidney transplantation could be an opportunity for this population. Over the past 20 years, more and more experience has been gained worldwide with this technique and promising results have been achieved, particularly for overweight patients [7–9].

Intraoperatively, we found minor statistically significant differences regarding the technique of vascular anastomotic suturing. In general, there is no suturing technique for vascular anastomoses that is proven to be superior to others [10]. There is a unanimous opinion that the choice of suturing technique should depend on the surgeon’s preference and experience [10]. The differences shown here are therefore most likely not clinically relevant.

The insertion of a ureteral stent during kidney transplantation has also become standard practice. There were no statistically significant differences regarding this procedure. This is to be welcomed as the use of ureteral stents has been shown to significantly reduce the incidence of major urological complications such as urinary leaks and ureteral stenosis [11].

Last but not least, the insertion of a bladder catheter is a well-known perioperative standard in kidney transplantations. Surgical centers showed statistically significant shorter catheterization times compared to urological centers. Similar observations can be found in the UK: Amer et al. observed a mean catheterization time of 5 days [12]. In the UK only surgical centers perform kidney transplants.

In line with these findings, there are several studies which postulate an optimal catheterization duration of 4 to 5 days after transplantation, although some teams describe even shorter periods as safely feasible [13, 14]. From a urological perspective, the reason for a longer catheterization time is to avoid tension on the ureteral anastomosis and to facilitate continuous urine drainage, which helps to monitor perioperative fluid balance and reduces the risk of urinary leakage. However, it is important to weigh the benefits of prolonged catheterization against the increased risk of urinary tract infections and potentially prolonged hospitalization.

Limitations of the data available here result from the anonymity of the interviewees and a possible self-reporting bias.

## Conclusion

The majority of kidney transplants is performed according to similar standards in Germany, irrespective of the performing surgical (sub)specialty. There are minor differences in the individual specialists’ approach, e.g. regarding the technique of vascular anastomosis. Some special features should be scientifically evaluated.

## Supplementary Information

Below is the link to the electronic supplementary material.

Supplementary Material 1.

Supplementary Material 2.

## Data Availability

No datasets were generated or analysed during the current study.

## Abbreviations

e.g.: Exempli gratia
vs: Versus
Ch: Charrière
