# Supplementary material for: Current clinical standards for renal transplantation: a survey among urological and surgical transplantation centers in Germany
Source: World J Urol. 2025 Nov 20;43(1):710. doi: 10.1007/s00345-025-06094-2 (PMC12634771; doi:10.1007/s00345-025-06094-2)
Supplement: Supplementary file 1 — Supplementary Material 1. [file 345_2025_6094_MOESM1_ESM.docx]

# Survey on standards for kidney transplantation

The following questions serve to evaluate the different “house standards” that are used for kidney transplants. When answering the questions, please think about the typical course of an unremarkable procedure as it is usual in your clinic.

1. In which center for kidney transplants do you work? Please name the clinic and the location.

2. In which department do you work?

O Clinic for Urology

O Clinic for Nephrology

O Clinic for General Surgery

O other

3. Which technique is used in your clinic for kidney transplantation? (multiple answers possible)

O hockey stick incision – transperitoneal

O hockey stick incision – extraperitoneal

O median laparotomy

O Pararectal incision

O Laparoscopic

O Robot-assisted laparoscopic

O other

4. Which retractor is used in your clinic for kidney transplantation?

O Omni-Tract® Retractor

O Sealing frame Retractor

O Hegemann Retractor

O Finochietto Retractor

O Bookwalter Retractor

O other

5. Is the peritoneum opened intraoperatively during kidney transplantation for lymphoceles prophylaxis?

O Yes

O No

6. What technique is used in your clinic for ureteral anastomosis as part of kidney transplantation?

O Lich-Gregoir

O Leadbetter-Politano

O Cohen

O Fullthickness technique

O other

7. Is a separate anti-reflux plastic surgery performed as part of the kidney transplant?

O Yes

O No

8. Is a drain inserted intraoperatively during kidney transplantation?

O Yes

O No

9. In the case of a drainage system - Which drainage system is used in your clinic for kidney transplantation?

O no drain

O 15 Ch drain

O 18 Ch drain

O 21 Ch drain

O other

10. Is a laboratory determination of creatinine from the drainage secretion carried out before the drainage is removed?

O Yes, in any case.

O Individual decision.

O No examination of the secretion.

O No drainage is inserted.

11. Does your clinic install an ureteral stent as part of the kidney transplant? If so, what size is used?

O no Ureteral stent

O 6 Ch Ureteral stent

O 7 Ch Ureteral stent

O 8 Ch Ureteral stent

O other

12. How long is the inserted ureteral stent usually left in place in your clinic for patients after kidney transplantation?

O 14 days

O 21 days

O < 21 days

O no ureteral stent

13. Which bladder catheter is used in your clinic for kidney transplantation?

O 16 Ch Bladder catheter

O 18 Ch Bladder catheter

O 20 Ch Bladder catheter

O other

14. Is a cystogram performed before the bladder catheter is removed in routine cases?

O Yes

O No

15. How long is the inserted bladder catheter usually left in place in your clinic for patients after kidney transplantation?

O < 7 days

O 7 – 10 days

O 11 – 21 days

O > 21 days

16. Does your clinic administer intraoperative heparin during kidney transplantation? If yes, in what dosage?

O No intraoperative administration of heparin

O Yes, 1500 IU heparin

O Yes, 1500 – 2000 IU heparin

O Yes, > 2000 IU heparin

O other

17. Does your clinic administer intraoperative diuretics during kidney transplantation?

O Yes

O No

18. Does your clinic administer intraoperative mannitol during kidney transplantation?

O Yes

O No

19. Does your clinic perform intraoperative sonography as standard during kidney transplantation?

O Yes, sonography before fascial closure

O Yes, sonography after skin closure

O No standard sonography

20. Does your clinic perform an intraoperative renal biopsy as part of kidney transplantation?

O Yes

O No

21. What type of wound dressing is used in your clinic after skin suturing following kidney transplantation?

O Plaster dressing (white)

O Closure with transparent film

O other

22. Which suture material is usually used in your clinic for suturing arterial vascular anastomoses? (Please state the type of suture and the suture thickness).

23. Which suture material is usually used in your clinic for suturing venous vascular anastomoses? (Please state the type of suture and the suture thickness).

24. Which suture material is usually used in your clinic for suturing ureteral anastomoses? (Please state the type of suture and the suture thickness).

25. Which technique is used in your clinic to suture the arterial vascular anastomosis during kidney transplantation? (multiple answers possible)

O Continuous in parachute technique

O Continuous, by quadrant

O Continuous, by front and back

O Single button seam

O other

26. Which technique is used in your clinic to suture the venous vascular anastomosis during kidney transplantation? (multiple answers possible)

O Continuous in parachute technique

O Continuous, by quadrant

O Continuous, by front and back

O Single button seam

O other

27. To which vessel is the transplant artery usually connected during kidney transplantation?

O A. iliaca externa

O A. iliaca interna

O A. iliaca communis

O other

28. To which vessel is the transplant vein usually connected during kidney transplantation?

O V. iliaca externa

O V. iliaca interna

O V. iliaca communis

O other

29. Do you carry out living kidney donations in your clinic?

O Yes

O No

30. Which imaging (donor) is performed pre-operatively in preparation for a living kidney donation in your clinic? (multiple answers possible)

O Sonography

O CT abdomen native

O CT angio

O MRI abdomen

O other

31. Up to what number of arterial vessels of the donor kidney can be used as a donor organ (in the context of living kidney donation) in your clinic?

O 2

O 3

O 4

O 5

O other

32. How does the removal of the donor organ in the context of a living kidney donation normally take place in your clinic? (multiple answers possible)

O lumbar incision

O transperitoneal incision

O laparoscopic

O robot-assisted laparoscopic

O other

33. How is the donor's artery usually closed in the context of a living kidney donation?

O stapler / GIA

O Metal clip

O Hem-o-lock + suture

O Hem-o-lock + ligature

O Hem-o-lock alone

34. How is the donor's vein usually closed in the context of a living kidney donation?

O stapler / GIA

O Metal clip

O Hem-o-lock + suture

O Hem-o-lock + ligature

O Hem-o-lock alone

35. Do you carry out pediatric kidney transplants in your clinic?

O Yes

O No

36. Does your clinic require patients to abstain from smoking before being listed for kidney transplantation?

O Yes

O No

37. Does your clinic have an obligation to reduce weight (for obese patients) before listing for kidney transplantation?

O Yes

O No

38. Does your clinic have a maximum body mass index (BMI) for patients before kidney transplantation? If yes, please state this.

O No

O Yes, maximum BMI …

39. Please enter the estimated mean body mass index (BMI) of the patients receiving a kidney transplant in your clinic.

40. Is a nephrectomy necessary before transplanting a patient with cystic kidneys?

O No

O Individual decision

O Yes

O Yes, if patient is symptomatic

41. Does your clinic perform transplant nephrectomies on pre-transplant patients prior to re-transplantation?

O Yes

O Individual decision

O No

42. Where are your patients cared for immediately after a kidney transplant?

O Intensive care unit

O intermediate care unit

O normal ward

43. Which department is primarily responsible for post-operative treatment?

O Nephrology

O Urology

O Surgery

O interdisciplinary concept

O other

44. Is bladder tenesmus treated with medication postoperatively?

O no drug treatment

O drug treatment with trospium chloride

O drug treatment with oxybutynin

O drug treatment with analgesics

45. Which department discharges patients after a kidney transplant?

O Nephrology

O Urology

O Surgery

O interdisciplinary concept

O other

46. When can patients be discharged?

47. How does your clinic manage post-operative urinary transport disorders?

O Insertion or change of ureteral stent

O Insertion of a percutaneous nephrostomy

O no invasive treatment

O other

48. How does your clinic manage post-operative symptomatic lymphocele?

O Insertion of a drain

O open surgical lymphocele fenestration

O laparoscopic surgical lymphocele fenestration

O no invasive treatment

O other

49. How does your clinic manage symptomatic post-operative reflux (e.g. in the case of recurrent urinary tract infections)?

O Injection of the ostium (e.g. Deflux ®)

O ureteral reimplantation

O no invasive treatment

O other
