# Supplementary material for: Current clinical standards for renal transplantation: a survey among urological and surgical transplantation centers in Germany
Source: World J Urol. 2025 Nov 20;43(1):710. doi: 10.1007/s00345-025-06094-2 (PMC12634771; doi:10.1007/s00345-025-06094-2)
Supplement: Supplementary file 2 — Supplementary Material 2. [file 345_2025_6094_MOESM2_ESM.docx]

# Additional Data on standards for kidney transplantation

3. Which technique is used in your clinic for kidney transplantation? (multiple answers possible)

|  | Urologists | General surgeons |
| --- | --- | --- |
| hockey stick incision – transperitoneal | 0 | 1 (4.8%) |
| hockey stick incision – extraperitoneal | 10 (76.9%) | 19 (90.5%) |
| median laparotomy | 2 (15.4%) | 0 |
| Pararectal incision | 1 (7.7%) | 0 |
| Laparoscopic | 0 | 0 |
| Robot-assisted laparoscopic | 3 (23.1%) | 1 (4.8%) |
| other | 0 | 0 |

4. Which retractor is used in your clinic for kidney transplantation?

|  | Urologists | General surgeons |
| --- | --- | --- |
| Omni-Tract® Retractor | 5 (38.5%) | 4 (19.0%) |
| Sealing frame Retractor | 1 (7.8%) | 0 |
| Hegemann Retractor | 0 | 0 |
| Finochietto Retractor | 0 | 3 (14.3%) |
| Bookwalter Retractor | 2 (15.4%) | 4 (19.0%) |
| other | 4 (30.8%) | 7 (33.3%) |
| not specified | 2 (15.4%) | 2 (9.5%) |

5. Is the peritoneum opened intraoperatively during kidney transplantation for lymphoceles prophylaxis?

|  | Urologists | General surgeons |
| --- | --- | --- |
| Yes | 3 (23.1%) | 1 (4.5%) |
| No | 9 (69.2%) | 18 (81.8%) |
| not specified | 1 (7.7%) | 3 (13.6%) |

6. What technique is used in your clinic for ureteral anastomosis as part of kidney transplantation?

|  | Urologists | General surgeons |
| --- | --- | --- |
| Lich-Gregoir | 11 (84.6%) | 16 (76.2%) |
| Leadbetter-Politano | 0 | 1 (4.8%) |
| Cohen | 0 | 0 |
| Fullthickness technique | 1 (7.7%) | 2 (9.5%) |
| not specified | 1 (7.7%) | 2 (9.5%) |

7. Is a separate anti-reflux plastic surgery performed as part of the kidney transplant?

|  | Urologists | General surgeons |
| --- | --- | --- |
| Yes | 1 (7.7%) | 13 (61.9%) |
| No | 11 (84.6%) | 5 (23.8%) |
| not specified | 1 (7.7%) | 3 (14.3%) |

8. Is a drain inserted intraoperatively during kidney transplantation?

|  | Urologists | General surgeons |
| --- | --- | --- |
| Yes | 11 (84.6%) | 16 (76.2%) |
| No | 0 | 2 (9.5%) |
| not specified | 2 (15.4%) | 3 (14.3%) |

9. In the case of a drainage system - Which drainage system is used in your clinic for kidney transplantation?

|  | Urologists | General surgeons |
| --- | --- | --- |
| 15 Ch drain | 1 (7.7%) | 2 (10%) |
| 16 Ch drain | 2 (15.4%) | 1 (5%) |
| 18 Ch drain | 0 | 8 (40%) |
| 21 Ch drain | 4 (30.8%) | 0 |
| other | 3 (23.1%) | 5 (25%) |
| not specified | 3 (23.1%) | 4 (20%) |

10. Is a laboratory determination of creatinine from the drainage secretion carried out before the drainage is removed?

|  | Urologists | General surgeons |
| --- | --- | --- |
| Yes | 4 (30.8%) | 3 (14.3%) |
| No | 2 (15.4%) | 5 (23.8%) |
| Individual decision | 6 (46.2%) | 11 (52.4%) |
| not specified | 1 (7.7%) | 2 (9.5%) |

11. Does your clinic install an ureteral stent as part of the kidney transplant? If so, what size is used?

|  | Urologists | General surgeons |
| --- | --- | --- |
| Yes, 6 Ch | 5 (38.5%) | 8 (38.1%) |
| Yes, 7 Ch | 7 (53.9%) | 7 (33.3%) |
| Yes, 8 Ch | 0 | 1 (4.8%) |
| No | 0 | 1 (4.8%) |
| not specified | 1 (7.7%) | 4 (19.0%) |

12. How long is the inserted ureteral stent usually left in place in your clinic for patients after kidney transplantation?

|  | Urologists | General surgeons |
| --- | --- | --- |
| 14 days | 1 (7.7%) | 2 (9.5%) |
| 21 days | 4 (30.8%) | 3 (14.3%) |
| > 21 days | 6 (46.2%) | 13 (61.9%) |
| not specified | 2 (15.4%) | 3 (14.3%) |

13. Which bladder catheter is used in your clinic for kidney transplantation?

|  | Urologists | General surgeons |
| --- | --- | --- |
| 16 Ch Bladder catheter | 9 (69.2%) | 10 (47.6%) |
| 18 Ch Bladder catheter | 0 | 6 (28.6%) |
| 20 Ch Bladder catheter | 2 (15.4%) | 1 (4.8%) |
| not specified | 2 (15.4%) | 4 (19.0%) |

14. Is a cystogram performed before the bladder catheter is removed in routine cases?

|  | Urologists | General surgeons |
| --- | --- | --- |
| Yes | 1 (7.7%) | 0 |
| No | 10 (76.9%) | 19 (90.5%) |
| not specified | 2 (15.4%) | 2 (9.5%) |

15. How long is the inserted bladder catheter usually left in place in your clinic for patients after kidney transplantation?

|  | Urologists | General surgeons |
| --- | --- | --- |
| < 7 days | 2 (15.4%) | 13 (61.9%) |
| 7 – 10 days | 8 (61.5%) | 6 (28.6%) |
| 11 – 21 days | 1 (7.7%) | 0 |
| not specified | 2 (15.4%) | 2 (9.5%) |

16. Does your clinic administer intraoperative heparin during kidney transplantation? If yes, in what dosage?

|  | Urologists | General surgeons |
| --- | --- | --- |
| Yes * | 4 (30.8%) | 1 (4.8%) |
| No | 7 (53.8%) | 18 (85.7%) |
| not specified | 2 (15.4%) | 2 (9.5%) |

* Dosage individual decision

17. Does your clinic administer intraoperative diuretics during kidney transplantation?

|  | Urologists | General surgeons |
| --- | --- | --- |
| Yes | 4 (30.8%) | 8 (38.1%) |
| No | 7 (53.8%) | 11 (52.4%) |
| not specified | 2 (15.4%) | 2 (9.5%) |

18. Does your clinic administer intraoperative mannitol during kidney transplantation?

|  | Urologists | General surgeons |
| --- | --- | --- |
| Yes | 3 (23.1%) | 9 (42.9%) |
| No | 8 (61.5%) | 10 (47.6%) |
| not specified | 2 (15.4%) | 2 (9.5%) |

19. Does your clinic perform intraoperative sonography as standard during kidney transplantation?

|  | Urologists | General surgeons |
| --- | --- | --- |
| Yes, sonography before fascial closure | 3 (23.1%) | 4 (19.0%) |
| Yes, sonography after skin closure | 1 (7.7%) | 6 (28.6%) |
| No standard sonography | 6 (46.2%) | 8 (38.1%) |
| not specified | 3 (23.1%) | 3 (14.3%) |

20. Does your clinic perform an intraoperative renal biopsy as part of kidney transplantation?

|  | Urologists | General surgeons |
| --- | --- | --- |
| Yes | 2 (15.4%) | 4 (19.0%) |
| No | 9 (69.2%) | 14 (66.6%) |
| not specified | 2 (15.4%) | 3 (14.3%) |

21. What type of wound dressing is used in your clinic after skin suturing following kidney transplantation?

|  | Urologists | General surgeons |
| --- | --- | --- |
| Plaster dressing (white) | 9 (69.2%) | 17 (81.0%) |
| Closure with transparent film | 0 | 1 (4.8%) |
| other | 2 (15.4%) | 1 (4.8%) |
| not specified | 2 (15.4%) | 2 (9.5%) |

22. Which suture material is usually used in your clinic for suturing arterial vascular anastomoses? (Please state the type of suture and the suture thickness).

|  | Urologists | General surgeons |
| --- | --- | --- |
| Prolene 5-0 | 9 (69.2%) | 8 (38.1%) |
| Prolene 6-0 | 0 | 8 (38.1%) |
| PDS 5-0 | 1 (7.7%) | 0 |
| PDS 6-0 | 0 | 1 (4.8%) |
| not specified | 3 (23.1%) | 4 (19.0%) |

23. Which suture material is usually used in your clinic for suturing venous vascular anastomoses? (Please state the type of suture and the suture thickness).

|  | Urologists | General surgeons |
| --- | --- | --- |
| Prolene 5-0 | 8 (61.5%) | 9 (42.9%) |
| Prolene 6-0 | 1 (7.7%) | 7 (33.3%) |
| PDS 5-0 | 1 (7.7%) | 1 (4.8%) |
| PDS 6-0 | 0 | 0 |
| not specified | 3 (23.1%) | 4 (19.0%) |

24. Which suture material is usually used in your clinic for suturing ureteral anastomoses? (Please state the type of suture and the suture thickness).

|  | Urologists | General surgeons |
| --- | --- | --- |
| PDS 5-0 | 6 (46.2%) | 6 (28.6%) |
| PDS 6-0 | 0 | 5 (23.8%) |
| PDS 4-0 | 0 | 2 (9.5%) |
| Vicryl 4-0 | 1 (7.7%) | 0 |
| Monocryl 4-0 | 0 | 1 (4.8%) |
| Monocryl 5-0 | 3 (23.1%) | 0 |
| not specified | 3 (23.1%) | 7 (33.3%) |

25. Which technique is used in your clinic to suture the arterial vascular anastomosis during kidney transplantation? (multiple answers possible)

|  | Urologists | General surgeons |
| --- | --- | --- |
| Continuous, parachute | 1 (7.7%) | 9 (42.9%) |
| Continuous, by quadrant | 2 (15.4%) | 0 |
| Continuous, by front and back | 8 (61.5%) | 12 (57.1%) |
| Single button seam | 2 (15.4%) | 2 (9.5%) |

26. Which technique is used in your clinic to suture the venous vascular anastomosis during kidney transplantation? (multiple answers possible)

|  | Urologists | General surgeons |
| --- | --- | --- |
| Continuous, parachute | 0 | 6 (28.6%) |
| Continuous, by quadrant | 3 (23.1%) | 1 (4.8%) |
| Continuous, by front and back | 7 (53.8%) | 11 (52.4%) |
| Single button seam | 0 | 0 |

27. To which vessel is the transplant artery usually connected during kidney transplantation?

|  | Urologists | General surgeons |
| --- | --- | --- |
| A. iliaca externa | 10 (76.9%) | 11 (52.4%) |
| A. iliaca communis | 0 | 6 (28.6%) |
| not specified | 3 (23.1%) | 4 (19.0%) |

28. To which vessel is the transplant vein usually connected during kidney transplantation?

|  | Urologists | General surgeons |
| --- | --- | --- |
| A. iliaca externa | 10 (76.9%) | 14 (66.6%) |
| A. iliaca communis | 0 | 1 (4.8%) |
| V. cava | 0 | 3 (14.3%) |
| not specified | 3 (23.1%) | 3 (14.3%) |

29. Do you carry out living kidney donations in your clinic?

|  | Urologists | General surgeons |
| --- | --- | --- |
| Yes | 11 (84.6%) | 18 (85.7%) |
| No | 0 | 0 |
| not specified | 2 (15.4%) | 3 (14.3%) |

30. Which imaging (donor) is performed pre-operatively in preparation for a living kidney donation in your clinic? (multiple answers possible)

|  | Urologists | General surgeons |
| --- | --- | --- |
| Sonography | 7 (53.8%) | 8 (38.1%) |
| CT abdomen native | 1 (7.7%) | 2 (9.5%) |
| CT angio | 6 (46.1%) | 10 (47.6%) |
| MRI abdomen | 4 (30.8%) | 9 (42.9%) |
| other | 2 (15.4%) | 1 (4.8%) |

31. Up to what number of arterial vessels of the donor kidney can be used as a donor organ (in the context of living kidney donation) in your center?

|  | Urologists | General surgeons |
| --- | --- | --- |
| 2 | 5 (38.5%) | 5 (23.8%) |
| 3 | 5 (38.5%) | 10 (47.6%) |
| individual decision | 0 | 2 (9.5%) |
| not specified | 3 (23.1%) | 4 (19.0%) |

32. How does the removal of the donor organ in the context of a living kidney donation normally take place in your center? (multiple answers possible)

|  | Urologists | General surgeons |
| --- | --- | --- |
| lumbar incision | 6 (46.1%) | 1 (4.8%) |
| transperitoneal incision | 0 | 1 (4.8%) |
| laparoscopic | 4 (30.8%) | 12 (57.1%) |
| robot-assisted laparoscopic | 1 (7.7%) | 1 (4.8%) |
| other | 0 | 5 (23.8%) |

33. How is the donor's artery usually closed in the context of a living kidney donation?

|  | Urologists | General surgeons |
| --- | --- | --- |
| stapler / GIA | 1 (7.7%) | 12 (57.1%) |
| Metal clip | 0 | 4 (19.0%) |
| Hem-o-lock alone | 3 (23.1%) | 1 (4.8%) |
| Hem-o-lock + suture | 5 (38.5%) | 1 (4.8%) |
| ligature | 1 (7.7%) | 0 |
| not specified | 3 (23.1%) | 3 (14.3%) |

34. How is the donor's vein usually closed in the context of a living kidney donation?

|  | Urologists | General surgeons |
| --- | --- | --- |
| stapler / GIA | 4 (30.8%) | 13 (61.9%) |
| Metal clip | 0 | 1 (4.8%) |
| Hem-o-lock alone | 0 | 1 (4.8%) |
| Hem-o-lock + suture | 2 (15.4%) | 1 (4.8%) |
| ligature | 4 (30.8%) | 2 (9.5%) |
| not specified | 3 (23.1%) | 3 (14.3%) |

35. Do you carry out pediatric kidney transplants in your hospital?

|  | Urologists | General surgeons |
| --- | --- | --- |
| Yes | 5 (38.5%) | 7 (33.3%) |
| No | 6 (46.2%) | 11 (52.4%) |
| not specified | 2 (15.4%) | 3 (14.3%) |

36. Does your clinic require patients to abstain from smoking before being listed for kidney transplantation?

|  | Urologists | General surgeons |
| --- | --- | --- |
| Yes | 1 (7.7%) | 5 (23.8%) |
| No | 10 (76.9%) | 11 (52.4%) |
| not specified | 2 (15.4%) | 5 (23.8%) |

37. Does your clinic have an obligation to reduce weight (for obese patients) before listing for kidney transplantation?

|  | Urologists | General surgeons |
| --- | --- | --- |
| Yes | 4 (30.8%) | 11 (52.4%) |
| No | 7 (53.8%) | 5 (23.8%) |
| not specified | 2 (15.4%) | 5 (23.8%) |

38. Does your clinic have a maximum body mass index (BMI) for patients before kidney transplantation?

|  | Urologists | General surgeons |
| --- | --- | --- |
| Yes | 4 (30.8%) | 13 (61.9%) |
| No | 7 (53.8%) | 3 (14.3%) |
| not specified | 2 (15.4%) | 5 (23.8%) |

39. Please enter the estimated mean body mass index (BMI) of the patients receiving a kidney transplant in your center.

Urologist: median 35 [30 – 39]

General surgeons: median 35 [30 – 40]

40. Is a nephrectomy necessary before transplanting a patient with cystic kidneys?

|  | Urologists | General surgeons |
| --- | --- | --- |
| Yes | 2 (15.4%) | 4 (19.0%) |
| No | 0 | 1 (4.8%) |
| Individual decision | 9 (69.2%) | 12 (57.1%) |
| not specified | 2 (15.4%) | 4 (19.0%) |

41. Does your clinic perform transplant nephrectomies on pre-transplant patients prior to re-transplantation?

|  | Urologists | General surgeons |
| --- | --- | --- |
| Yes | 0 | 0 |
| No | 3 (23.1%) | 1 (4.8%) |
| Individual decision | 8 (61.5%) | 16 (76.2%) |
| not specified | 2 (15.4%) | 4 (19.0%) |

42. Where are your patients cared for immediately after a kidney transplant?

|  | Urologists | General surgeons |
| --- | --- | --- |
| Intensive care unit | 3 (23.1%) | 6 (28.6%) |
| intermediate care unit | 6 (46.2%) | 6 (28.6%) |
| normal ward | 2 (15.4%) | 5 (23.8%) |
| not specified | 2 (15.4%) | 4 (19.0%) |

43. Which department is primarily responsible for post-operative treatment?

|  | Urologists | General surgeons |
| --- | --- | --- |
| Nephrology | 4 (30.8%) | 3 (14.3%) |
| Urology/Surgery | 6 (46.2%) | 4 (19.0%) |
| interdisciplinary concept | 1 (7.7%) | 10 (47.6%) |
| not specified | 2 (15.4%) | 4 (19.0%) |

44. Is bladder tenesmus treated with medication postoperatively?

|  | Urologists | General surgeons |
| --- | --- | --- |
| no drug treatment | 3 (23.1%) | 10 (47.6%) |
| drug treatment with trospium chloride or oxybutynin | 2 (15.4%) | 2 (9.5%) |
| drug treatment with analgesics | 5 (38.5%) | 4 (19.0%) |
| not specified | 3 (23.1%) | 5 (23.8%) |

45. Which department discharges patients after a kidney transplant?

|  | Urologists | General surgeons |
| --- | --- | --- |
| Nephrology | 6 (46.2%) | 7 (33.3%) |
| Urology/Surgery | 4 (30.8%) | 8 (38.1%) |
| interdisciplinary concept | 1 (7.7%) | 2 (9.5%) |
| not specified | 2 (15.4%) | 4 (19.0%) |

46. When can patients be discharged?

|  | Urologists | General surgeons |
| --- | --- | --- |
| 6 – 8 days | 0 | 5 (23.8%) |
| 10 – 14 days | 10 (76.9%) | 9 (42.9%) |
| 21 days + | 1 (7.7%) | 3 (14.3%) |
| not specified | 2 (15.4%) | 4 (19.0%) |

47. How does your clinic manage post-operative urinary transport disorders?

|  | Urologists | General surgeons |
| --- | --- | --- |
| Insertion or change of ureteral stent | 8 (61.5%) | 12 (57.1%) |
| Insertion of a percutaneous nephrostomy | 0 | 2 (9.5%) |
| Individual decision | 3 (23.1%) | 2 (9.5%) |
| not specified | 2 (15.4%) | 5 (23.8%) |

48. How does your clinic manage post-operative symptomatic lymphocele?

|  | Urologists | General surgeons |
| --- | --- | --- |
| Insertion of a drain | 7 (53.8%) | 9 (42.9%) |
| open surgical lymphocele fenestration | 0 | 0 |
| laparoscopic surgical lymphocele fenestration | 3 (23.1%) | 7 (33.3%) |
| Individual decision | 1 (7.7%) | 1 (4.8%) |
| not specified | 2 (15.4%) | 4 (19.0%) |

49. How does your clinic manage symptomatic post-operative reflux (e.g. in the case of recurrent urinary tract infections)?

|  | Urologists | General surgeons |
| --- | --- | --- |
| no invasive treatment | 2 (15.4%) | 2 (9.5%) |
| Injection of the ostium  (e.g. Deflux ®) | 1 (7.7%) | 1 (4.8%) |
| ureteral reimplantation | 4 (30.8%) | 12 (57.1%) |
| Individual decision | 4 (30.8%) | 1 (4.8%) |
| not specified | 2 (15.4%) | 5 (23.8%) |
